# Microbial Primer: Phase variation - survival and adaptability by generation of a diverse population

**DOI:** 10.1099/mic.0.001492

**Published:** 2024-09-02

**Authors:** Ashley J. Fraser, Finn E. McMahon, John M. Atack

**Affiliations:** 1 Institute for Biomedicine & Glycomics, Griffith University, Gold Coast, Queensland 4215, Australia; 2 School of Environment and Science, Griffith University, Gold Coast, Queensland 4215, Australia

**Keywords:** contingency/survival strategy, inverted repeats, phase variation, phasevarion, simple sequence repeats

## Abstract

Phase variation is defined as the rapid and reversible switching of gene expression, and typically occurs in genes encoding surface features in small genome bacterial pathogens. Phase variation has evolved to provide an extra survival mechanism in bacteria that lack multiple ‘sense-and-respond’ gene regulation systems. Many bacterial pathogens also encode DNA methyltransferases that are phase-variable, controlling systems called ‘phasevarions’ (phase-variable regulons). This primer will summarize the current understanding of phase variation, describing the role of major phase-variable factors, and phasevarions, in bacterial pathobiology.

## Introduction

Phase variation is the rapid and reversible switching of gene expression [1]. It is typically associated with genes encoding bacterial surface features, such as adhesins, pili, iron acquisition proteins, lipo-oligosaccharide (LOS), and lipopolysaccharide (LPS). Phase variation allows a population of organisms to generate a variety of phenotypic variants, with these mixed populations containing individuals that are, for example, better equipped to colonize certain host niches, or primed to evade an immune response. Phase-variable genes are easily identified from the genomic DNA sequence of an organism, as they contain a number of well-defined DNA sequence features. These DNA sequence features are simple sequence repeat (SSR) tracts and inverted repeats (IRs). Several bacterial pathogens contain multiple phase-variable genes, with the suite of phase-variable genes in a particular species referred to as a ‘phasome’ [2].

**Fig. 1.**
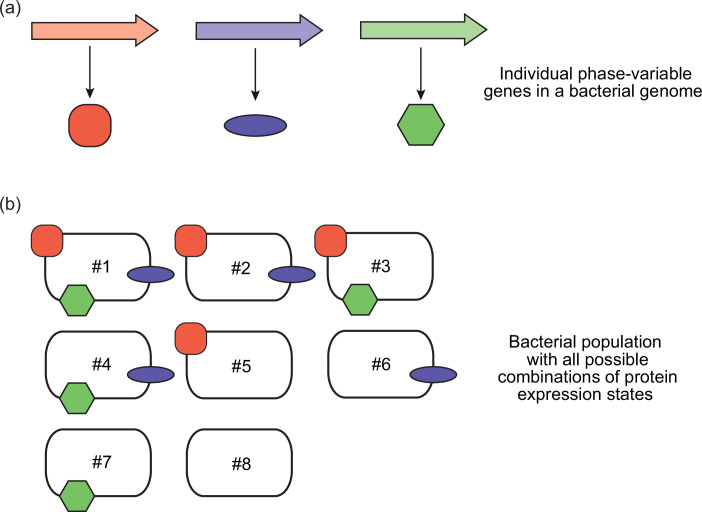
The whole is greater than the sum of its parts - multiple phase-variable genes in combination can generate a wide variety of phenotypes. The power of phase variation comes from the multiple combinations of protein expression profiles possible in combination when multiple phase-variable genes are encoded by a strain/species. In this example, (**a**) just three genes varying their expression ON–OFF results in (b) eight different phenotypes (2^3^=8) in a bacterial population – individual bacterial cells can express all three proteins (all genes are ON; phenotype 1 – #1), none of the proteins are expressed (all genes are OFF; phenotype 8 – #8) and all combinations in between. Therefore, if, for example, a strain encoded ten phase-variable genes all able to switch their expression ON or OFF, then there would be 2^10^ phenotypes possible in a population (1024 phenotypes). If multiple expression states are possible (not just ON or OFF), then the number of phenotypes possible increases even further from just a small set of phase-variable genes.

The power of phase-variable genes in generating diversity derives from the combination of expression states possible when a strain encodes multiple genes all able to vary their expression (Fig. 1). Although the precise numbers of phase-variable genes present in individual bacterial species can vary depending on the strain(s) studied and the method used to classify a gene as phase-variable, they can range from fewer than ten (e.g. *Escherichia coli, Pseudomonas aeruginosa*) to over 50 (*Neisseria meningitidis*). If all these phase-variable genes are just switching between two expression states in *N. meningitidis*, then there are theoretically >10^14^ different phenotypic states possible in a population.

Many bacterial pathogens also encode methyltransferase genes, associated with restriction-modification systems, that are subject to phase variation. Phase variation of a DNA methyltransferase results in variable genome-wide methylation within a bacterial population. This variable methylation leads to altered expression of multiple genes through epigenetic mechanisms. These systems are called phasevarions (phase-variable regulons). All described phasevarions regulate expression of multiple genes, including genes that are involved in host colonization, survival and pathogenesis, and many regulate putative vaccine candidates. The identification of phase-variable methyltransferases is straightforward – they also contain either SSR tracts or IRs. However, the genes regulated by them *do not* contain any easily identifiable features, and the conditions influencing the expression of genes controlled by phasevarions is not well defined. Phasevarions therefore provide an extra contingency strategy to survive changing environmental conditions. The only way to identify genes in a phasevarion is by detailed study of the organisms containing such systems.

## Mechanisms of phase variation

**Fig. 2.**
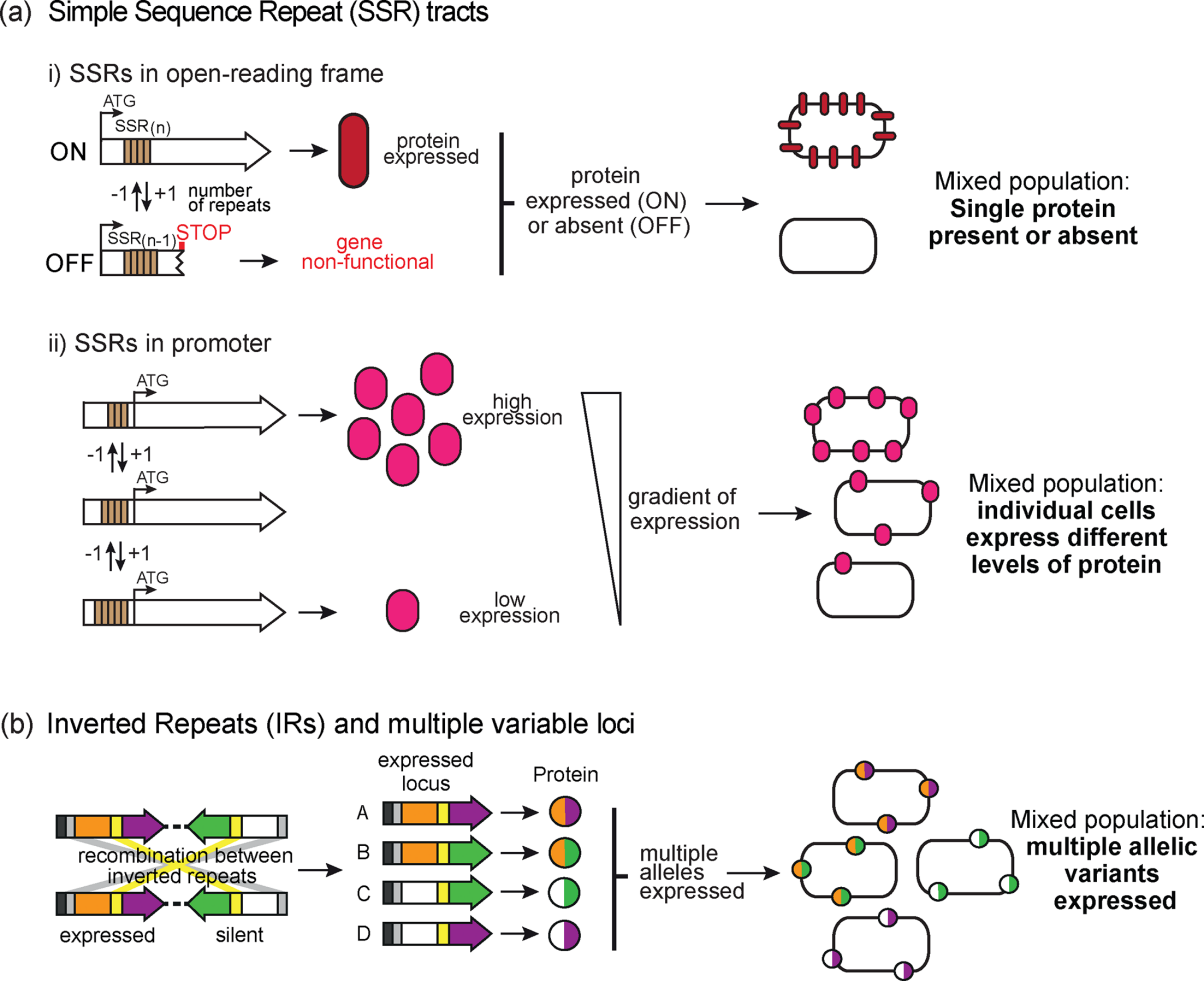
Illustration of the two major mechanisms leading to phase-variable gene expression. (**a**) Variation in the number of simple sequence repeats (SSRs; orange boxes) associated with a gene: (i) if the SSR tract is located in the ORF of a gene, this results in ON–OFF switching of expression of the encoded protein. If the number of SSRs leads to the gene remaining in-frame, the gene is expressed (ON). Gain or loss of repeat units in the SSR tract results in a frame-shift downstream of the SSR tract, and a premature stop codon, meaning the gene is non-functional (OFF). The bacterial population therefore contains variants that express the protein (ON), or that do not (OFF). (ii) If an SSR tract is located in the promoter of a gene, this leads to a gradient of protein expression, from high to low expression, commensurate with the length of the SSR tract. Longer tracts typically lead to lower protein expression. The resulting bacterial population therefore contains a mixture of variants. (**b**) Phase-variable loci can shuffle between expressed (orange+purple) and silent (white+green) loci, often by recombination between inverted repeats (IRs; yellow and grey boxes). In this example, each gene encodes a protein with variable domains – there are two variable N-terminal domains (orange or white) and two different C-terminal domains (purple or green), meaning four different protein allelic variants can be expressed. The resulting bacterial population will express all four protein variants.

Genes can phase-vary by two key mechanisms (Fig. 2): (a) variation in length of hypermutable SSR tracts, and (b) recombination, or shuffling, between IRs contained in expressed and silent loci.

*SSR tracts* are unstable and vary in length through polymerase slippage during replication. Longer SSR tracts exhibit higher rates of phase variation. If an SSR tract is located in the ORF of a gene, variation in tract length can result in expression of the gene (ON), or due to a frame-shift mutation downstream of the SSR resulting in a premature stop codon, the gene is not expressed (OFF), or in some cases, a truncated protein is expressed. SSR tracts can also be located in the promoter of a gene, where they result in a gradient (high to low) of protein expression. SSRs tracts that lead to phase-variable expression of a gene are not divisible by three (T_n_, GA_n_, AGCC_n_, etc). Genes containing SSR tracts, also referred to as ‘hypermutable loci’, typically experience mutation rates of ~1×10^−3^ mutations per cell division, compared to the standard background mutation rate in bacteria (~1×10^−9^ per gene per cell division).

*Recombination between expressed and silent variants* of a particular locus result in expression of multiple allelic variants of a single protein. This often occurs via recombination between IRs that are present within these loci. Recombination between these IRs is often catalysed by a recombinase associated with the phase-variable locus; searching bioinformatically for recombinases is often a useful strategy to identify phase-variable genes, in addition to searching for SSRs or IRs. This type of phase-variable gene expression is also referred to as antigenic variation.

Genes can also phase-vary by several other mechanisms, including the inversion of promoter sequences, differential methylation and insertion/excision of transposable DNA elements.

## Phase-variable genes are often highly immunogenic

Phase-variable genes are often found on the cell surface, and as such make ideal targets for the immune system, and often raise a strong immune response. Therefore, evolution of the ability to ‘turn-off’ or vary the protein sequence expressed seems to involve the ability to evade the immune system and potentiate bacterial survival [3,4]. Phase-variable expression of genes also means that the proteins they encode are often discounted as vaccine candidates; unstable expression by decreasing or completely turning off production of a protein, or shuffling to a new allelic variant, means that the target of a vaccine is no longer there. This could result in loss of efficacy, or complete failure of a vaccine. However, phase-variable genes *can* be used as vaccine antigens as they are highly immunogenic, are located on the bacterial cell surface and are often highly conserved between strains. It is also reasonable to assume that phase-variable genes persist as they are required for some key stage of the bacterial lifestyle; evolution implies that if they were not required for a key stage of host colonization or disease, they would be dispensed with, and that phase variation of these genes evolved to avoid an immune response during non-essential conditions. Therefore, if their expression profile and role during colonization and disease is thoroughly understood, they can be included in rationally designed subunit vaccines. For example, the phase-variable NadA protein forms part of the 4c-MenB (Bexsero) vaccine against *N. meningitidis* serogroup B. This protein is highly expressed during invasive meningococcal infection, meaning that Bexsero targets an antigen that is essential for a key stage of disease.

## Phase variation of adhesins leads to key differences in colonization and disease

Non-typeable *Haemophilus influenzae* (NTHi) encodes several adhesins that are all phase-variable. For example, the HMW1 and HMW2 adhesins contain a heptanucleotide TCTTTCA_(n)_ SSR tract in their promoter regions. Increasing the number of TCTTTCA_(n)_ repeats results in lower HMW1/2 expression, which results in lower biofilm formation and a decrease in virulence [5]. HMW1/2 are also required for binding to related host cell receptors in the human airway. The HMW proteins are highly immunogenic and are currently being investigated as candidates for a vaccine against NTHi. A second NTHi adhesin, Hia, contains a T_(n)_ SSR tract in its promoter, and switches between high and low expression levels due to variation in length of this tract. Selection for high Hia expression occurs during colonization of the host nasopharynx, but as Hia is immunogenic, T_(n)_ tract lengths that result in gradually lower Hia protein expression levels are selected for during *in vitro* opsonophagocytic killing assays. Thus, a back-and-forth selection and counter-selection for Hia protein expression levels occurs during NTHi colonization and pathogenesis. A third NTHi adhesin, Lav, encoded by approximately two-thirds of NTHi strains, switches expression ON–OFF, commensurate with changes in the number of GCAA repeats in an SSR tract in the ORF of the encoding *lav* gene. ON–OFF switching of Lav results in key differences in adherence and invasion of human cells; when Lav is ON, NTHi forms a much larger biofilm, implying that this adhesin has a key role in a major pathology of multiple NTHi infections.

*Neisseria* species are one of the best studied models of a species encoding multiple phase-variable adhesins. Phase-variable adhesins in *Neisseria* switch expression ON–OFF, vary between high and low expression states, and are expressed as multiple allelic variants. In *Neisseria gonorrhoeae*, the major outer-membrane protein Opa is expressed as several different allelic variants due to changes in length of CTCTT_(n)_ SSR tracts in the ORF of multiple variable copies of the *opa* genes found in the *N. gonorrhoeae* genome. The selective pressure of the host immune system drives variation of Opa protein expression allowing evasion of pre-primed immune responses against this organism. In the closely related organism *N. meningitidis,* the promoter for the adhesin NadA contains a TAAA_(n)_ SSR tract, with the number of TAAA_(n)_ repeats affecting the spacing of key regulatory elements, resulting in variable expression. NadA has been shown to be highly expressed during invasive meningococcal infection [6], and NadA is a component of the current vaccine licensed against *N. meningitidis*, Bexsero (as discussed above). Interestingly, in the majority of strains isolated from patients who had received the Bexsero vaccine against *N. meningitidis*, the TAAA_(n)_ SSR repeat had switched to lengths that would result in low levels of NadA expression, implying selection for those strains primed to avoid the immune response elicited by this component of the vaccine. Thus, just like Hia in NTHi, different expression states of NadA occur during colonization and disease. NadA expression in isolates from patients vaccinated with BexSero needs to be closely monitored to ensure continued vaccine efficacy.

## Pili in the pathogenic *Neisseria* phase-vary by multiple mechanisms

Pili are key bacterial factors in host colonization, and major bacterial surface features. As such they are prime targets for an immune response, and have evolved to vary both their expression and sequence to avoid this. For example, expression of *pilC*, encoding the Type IV pili tip adhesin, switches ON–OFF by variation in length of a G_(n)_ SSR tract in the encoding gene. The major pili protein subunit, encoded by *pilE,* shuffles between multiple allelic variants by recombining with silent variable *pilS* loci: * N. gonorrhoeae* has one expressed *pilE* gene and up to 19 silent variable *pilS* genes, distributed in multiple regions throughout the *N. gonorrhoeae* genome; *N. meningitidis* typically encodes four to eight variable *pilS* sequences, contained in a single locus on the chromosome. Shuffling between *pilE* and *pilS* loci in *Neisseria* can also lead to non-functional sequences in the *pilE* locus, meaning cells phase-vary ON–OFF their pilus expression. RecA, involved in DNA recombination, is also essential for pilin antigenic variation in both *N. gonorrhoeae* and *N. meningitidis*. Variable colony formation in a population of *N. gonorrhoeae* results from variable expression of pili, which leads to differences in biofilm formation. The key role of pili in neisserial pathogenesis, and the fact that the *Neisseria* have evolved multiple mechanisms to vary both expression and sequence of this key structure, demonstrates the power of phase variation in the generation of diversity and survival in bacteria

## Iron – a key nutrient acquired by phase-variable surface proteins

Both *Haemophilus influenzae* and *N. meningitidis* encode multiple haemoglobin receptors that are phase-variable. In *Haemophilus influenzae*, genes for the related proteins HgpA, HgpB and HgbC contain CCAA_(n)_ SSR tracts in their ORFs, and switch their expression ON–OFF. HgpA expression is required for full virulence in an infant rat model of invasive disease, meaning selection for HgpA ON would probably occur in invasive *Haemophilus influenzae* isolates. In *N. meningitidis* the haemoglobin receptors HpuAB and HmbR also switch their expression ON–OFF, via variation in length of a G_(n)_ SSR tract. HmbR also shows high interstrain variation, with selection for different variants occuring *in vivo*. A meningococcal strain lacking both HpuAB and HmbR was less virulent in a rat model of infection, but not impaired in its growth in human blood. Both *hpuA* or *hmbR* were phase-varied ON in a collection of *in vivo* meningococcal isolates, just like the adhesin NadA, which would validate the use of these two key surface proteins as potential candidates in future vaccines to protect against *N. meningitidis*.

## Lipooligosaccharide – a highly variable surface glycan structure

LOS and LPS are major virulence factors in a number of bacterial pathogens; LPS consists of a core oligosaccharide plus an extended carbohydrate called the O-antigen, and is present in species such as *Salmonella* and *E. coli*; LOS consists of a core oligosaccharide only, and is present in small genome pathogens such as NTHi and the pathogenic *Neisseria*. This section will focus only on LOS phase variation. The biosynthetic enzymes, glycosyltransferases, that are responsible for building LOS in NTHi are encoded by phase-variable genes [7]. These genes all switch ON–OFF by variation in locus-encoded SSR tracts. Variation of the expression of these multiple enzymes results in a highly heterogenous LOS structure in an NTHi population. Selection for and against particular glycan moieties has been demonstrated to occur during multiple stages of NTHi pathobiology [8,9]. For example, Lic2A, which adds a galactose to LOS, is ON in human serum, as the addition of a galactose by the Lic2A enzyme is required for serum resistance, but Lic2A expression is switched OFF in the majority of invasive NTHi isolates, indicating a complex role for LOS modified by Lic2A. The galactose added by Lic2A is also a receptor for a bacteriophage that infects NTHi. Phase variants of NTHi with *lic2A* OFF are more resistant to phage infection, as they do not express the receptor for this bacteriophage.

*Campylobacter jejuni*, a major human gastric pathogen, also switches expression of many LOS biosynthetic enzymes via SSR tract variation in the encoding genes. Addition of terminal GM1 or GM2 gangliosides is dependent on variation in length of a G_(n)_ SSR tract in the *wlaN* gene, encoding a beta-1,3 galactosyltransferase. As these structures mimic host glycans, they allow immune evasion by *C. jejuni* but also result in the auto-immune disease Guillan-Barre syndrome seen in a small percentage of people who been infected by *C. jejuni*.

## Phasevarions – differential expression of multiple genes via phase variation of a DNA methyltransferase

Phase-variable regulons – phasevarions – control differential expression of multiple genes through phase variation of a single DNA methyltransferase [10]. This adds a further level of complexity to understanding gene expression in bacterial pathogens; although SSR tracts and inverted repeats are easily identifiable in the methyltransferase locus that is phase-variable, the genes controlled by the differential methylation resulting from phase variation of the methyltransferase are not – they contain no sequence features that can be identified *in silico*.

The best studied phasevarions are controlled by Mod DNA methyltransferases that switch their expression ON–OFF via SSR tracts located in the ORF of the encoding *mod* genes. It has been shown that *mod* genes control phasevarions in many host-adapted bacterial pathogens, including NTHi (ModA) [11], *N. gonorrhoeae* and *N. meningitidis* (ModA, ModB, ModD), *Helicobacter pylori* (ModH), *Streptococcus suis* (ModS) and *Actinobacillus pleuropneumoniae* (ModP and ModQ). In addition to ON–OFF switching of expression, *mod* genes are highly variable in the region responsible for the DNA sequence methylated by the encoded methyltransferase (the region encoding the target recognition domain, or TRD). This means that Mod proteins with different TRDs methylate different DNA targets (usually non-palindromic 5 bp sequences), and therefore control different phasevarions. For example, 22 different ModA alleles have been identified in NTHi [12] and *Neisseria* spp.; 17 different ModH alleles have been identified in *Helicobacter pylori*. Different *mod* genes (e.g. *modA* vs. *modH*) show very low sequence conservation. Different strains of the same species of bacteria typically only encode a single Mod protein controlling a phasevarion, but the allele encoded may not be the same as in another strain of the same species (e.g. nearly all strains of NTHi encode a *modA* gene, highly conserved in their 5′ and 3′ regions, but different strains encode different alleles to each other, such as *modA1* and *modA2*, all of which have a highly variable TRD). This means that these different strains have different phasevarions. Analysis of all *mod* genes present in the restriction enzyme database REBASE demonstrated that SSR tracts are present in ~20% of all *mod* genes, meaning these methyltransferases can potentially phase-vary, and therefore control a phasevarion. This indicates that ON–OFF switching of methyltransferases is a common and widespread strategy used by bacteria to generate phenotypic diversity and improve adaptability.

The SpnIII phasevarion in *Streptococcus pneumoniae* was the first described phasevarion controlled by phase variation of a methyltransferase that switches between multiple different variants [13]. The SpnIII methyltransferase shuffles between six different methyltransferase specificities through homologous recombination between multiple, variable methyltransferase loci that contain IRs. This is facilitated by, but not entirely dependent upon, multiple recombinases. This system is present in almost every strain of *S. pneumoniae*. The six methyltransferase specificities encoded by this system all methylate a different DNA target sequence, and consequently control a different phasevarion. Each individual bacterium only expresses one variant, meaning there are six different phasevarions expressed, and six different phenotypic variants in a population. Pneumococcal populations show highly variable phenotypes and gene expression profiles commensurate with which methyltransferase variant they express. The SpnIII phasevarion results in variable expression of capsule, a key virulence factor and the basis of current vaccines, and in variable expression of conserved protein antigens, being investigated as components of a universal pneumococcal vaccine. This adds a further level of complexity to pneumococcal pathobiology, and to designing a universal vaccine against this pathogen.

Although phasevarions have been characterized in multiple species, it is still not entirely clear how differential methylation by phase-variable methyltransferases affects gene expression. Methylation by a phase-variable methyltransferase leading to expression differences has only been demonstrated for two genes. In both these examples – the *flaA* gene controlled by the ModH5 phasevarion in *Helicobacter pylori*, and the *eda* gene controlled by the ModA11 phasevarion in *N. meningitidis –* this differential methylation leads to variable expression of these genes. This latter example in *N. meningitidis* nicely illustrates the complexity of phasevarion-regulated genes: *eda* is just one of 285 genes regulated in the ModA11 phasevarion in this organism. ModA11 methylates a specific DNA sequence (5′-ACGT^m6^AGG-3′), with this DNA sequence present in the promoter of the *eda* gene. Analysis of all 285 genes in the ModA11 phasevarion shows that this sequence is only present in the promoters of 26/285 genes regulated by this phasevarion; how the other 259 genes are regulated by differential methylation remains to be elucidated.

## Conclusions

Phase-variable genes have evolved to provide bacteria with extra contingency strategies to survive changing environmental conditions. This ranges from individual genes all varying their expression, to entire suites of genes being controlled by a single phase-variable regulator (phasevarions). The seeming ‘randomization’ of gene expression afforded by these mechanisms means detailed study of these systems is required to understand their role in the bacteria that encode them. As many phase-variable genes encode surface factors that are highly immunogenic, the evolution of this mechanism of gene expression seems obvious: immunoevasion. Nevertheless, these factors must be key to some part of the bacterial lifestyle, otherwise evolution would have dispensed with them entirely, as opposed to favouring variable expression. The study of these systems will be key to designing new and more effective vaccines, or improving existing vaccines, against the pathogens that contain them, particularly important with the increase in antibiotic resistance.

## Abbreviations

IR: inverted repeat
LOS: lipo-oligosaccharide
LPS: lipopolysaccharide
NTHi: non-typeable *Haemophilus influenzae*
SSR: simple sequence repeat
TRD: target recognition domain
